# Supplementary material for: A Likelihood Approach for Real-Time Calibration of Stochastic Compartmental Epidemic Models
Source: PLoS Comput Biol. 2017 Jan 17;13(1):e1005257. doi: 10.1371/journal.pcbi.1005257 (PMC5240920; doi:10.1371/journal.pcbi.1005257)
Supplement: S1 File — (TAR.GZ) [file pcbi.1005257.s014.tar.gz › HSPH_Online-SI-Revision/EnKF07/n1-mild/n1-mild_PF2_EnKF-summary2.pdf]

peak -8	3w spec	3w cum	5w spec	5w cum	end	3w spec LOG	3w cum LOG	5w spec LOG	5w cum LOG	end LOG
mean	380.2	833.3	119.6	429.1	78.	0.8	0.7	1.2	0.6	0.6
median	141.2	502.2	97.9	267.8	73.2	0.7	0.6	1.2	0.6	0.5
quantile .1	90.5	157.9	76.9	112.9	56.9	0.5	0.4	0.8	0.4	0.3
quantile .9	598.	1737.7	190.2	922.1	94.8	1.2	1.2	1.8	0.4	1.
42	26	31	30	38						

peak -4	3w spec	3w cum	5w spec	5w cum	end	3w spec LOG	3w cum LOG	5w spec LOG	5w cum LOG	end LOG
mean	115.2	203.8	95.	123.2	87.1	1.1	0.5	1.6	0.6	0.8
median	99.9	156.6	94.9	108.6	89.2	1.1	0.5	1.6	0.6	0.9
quantile .1	82.3	99.5	79.4	86.2	70.	0.6	0.3	1.2	0.4	0.3
quantile .9	156.4	425.4	106.2	191.9	100.1	1.4	0.7	2.	0.4	1.
26	45	11	43	31						

peak	3w spec	3w cum	5w spec	5w cum	end	3w spec LOG	3w cum LOG	5w spec LOG	5w cum LOG	end LOG
mean	121.5	152.5	173.6	130.1	102.1	1.1	0.5	1.5	0.6	0.8
median	114.1	132.4	102.9	116.1	96.7	1.2	0.5	1.5	0.6	0.8
quantile .1	87.3	101.9	92.	95.8	86.7	0.8	0.4	1.1	0.4	0.6
quantile .9	155.9	232.6	137.4	191.2	125.5	1.4	0.6	1.8	0.4	1.1
30	45	14	43	34						

peak +4	3w spec	3w cum	5w spec	5w cum	end	3w spec LOG	3w cum LOG	5w spec LOG	5w cum LOG	end LOG
mean	441.4	1973.9	845.9	2261.6	2673.4	1.	0.5	1.3	0.5	0.7
median	146.9	209.3	131.5	185.4	127.7	0.9	0.4	1.3	0.5	0.6
quantile .1	103.8	106.4	93.2	111.1	89.4	0.6	0.4	0.8	0.4	0.4
quantile .9	417.	392.3	1001.	342.2	352.	1.5	0.6	1.7	0.4	1.
41	47	30	47	39						

peak +8	3w spec	3w cum	5w spec	5w cum	end	3w spec LOG	3w cum LOG	5w spec LOG	5w cum LOG	end LOG
mean	2329.1	9762.6	1450.4	10 766.5	11 685.1	1.	0.8	1.	0.7	0.9
median	267.4	451.8	189.1	338.1	250.9	0.9	0.5	1.	0.5	0.7
quantile .1	95.7	185.5	96.6	129.6	91.	0.6	0.4	0.4	0.4	0.4
quantile .9	7394.	2437.	4480.	2426.	2734.	1.5	1.1	1.6	0.4	1.1
45	35	38	36	29	{}	{}	{}	{}	{}	{}
